# Equilibrium and Release Properties of Aqueous Dispersions of Non-Steroidal Anti-Inflammatory Drugs Complexed with Polyelectrolyte Eudragit E 100

**DOI:** 10.3797/scipharm.1107-17

**Published:** 2012-04-30

**Authors:** Daniela Alejandra Quinteros, Daniel Alberto Allemandi, Ruben Hilario Manzo

**Affiliations:** Departamento de Farmacia, Facultad de Ciencias Químicas, Universidad Nacional de Córdoba, Córdoba, CP 5000, Argentina

**Keywords:** Eudragit E100^®^, NSAID, Complexes, Drug delivery, Aqueous Dispersions

## Abstract

Equilibria and release properties of aqueous systems consisting of a set of five non-steroidal anti-inflammatory drugs (AH) complexed with the cationic polymethacrylate Eudragit E 100 (EU) are reported in this study. The composition (EU(AH)_50_ (HCl)_50_) having fifty mole percent of each counterion (A^−^ and Cl^−^) produces clear, stable aqueous dispersions in which a remarkably high proportion of AH (higher than 98%) is condensed with the PE under the form of ion pairs. This property expands the interval of pH in which AH are aqueous soluble. The set of AH contains members with and without an alpha methyl group (-(CH_3_)CH-COOH: Flurbiprofen, Naproxen, Ketoprofen) and (-CH_2_-COOH: Diclofenac, Indomethacin). The proportion of ion pairs in the complexes was lower in the former group. Release of AH from the complexes toward a saline (NaCl 0.9%) solution was assayed in Franz cells. The five complexes behaved as drug carriers that exhibited a slow drug release with a remarkable zero order. In line with the percentages of counterionic condensation observed, release rates from -(CH_3_)CH-COOH complexes were clearly higher than those of -CH_2_-COOH ones.

## Introduction

In previous papers we described the properties of aqueous dispersions of complexes obtained through the interaction between the cationic polymethacrylate Eudragit E 100 (EU) and model acid drugs (AH). Such complexes were also obtained as stable amorphous solids, in which the drugs are ionically attached to the polyelectolyte (PE) as was recognized by infrared spectroscopy (IR), X ray diffraction and differential scanning calorimetry (DSC) [1, 2]. Also reported was a solid dispersion of EU and the non-steroidal anti-inflammatory drug (NSAID) ibuprofen, obtained by hot mail extrusion, which was used to develop a tablet formulation [3]. Although in this case DSC and X ray diffraction revealed a molecular interaction, its ionic nature was not perceived by the authors because IR or other appropriate techniques were not performed. In the same way, in other reports [4] the ionic interaction EU-ibuprofen in a solid matrix subjected to dissolution was not highlighted. Therefore, the availability in literature of a solid knowledge of such interactions in the solid state as well as in aqueous dispersions is of relevance in the pharmaceutical field.

Eudragit E 100 is a cationic polyelectrolyte (PE) based on dimethylaminoethylmethacrylate and other neutral methacrylic acid esters. It is soluble in gastric fluid as well as in weakly acidic buffer solutions (up to approximately pH 5). This polymer, in its unprotonated form (EU), is soluble in organic solvents and insoluble in petroleum ether and water. It is currently used as coating in solid pharmaceutical dosage forms (for taste masking and protection) [5]. The information provided in [1, 2] has shown the remarkably high affinity between the protonated groups of the macroion EUH^+^ and anionic organic species *(A*^−^*)* that in aqueous systems generates the reversible association depicted in equation 1.

Eq. 1. $$\text{AH}+\text{EU}⇄A^{-}+{\text{EUH}}^{+}⇄[A^{-}{\text{EUH}}^{+}]$$

There, [*A*^−^EUH^+^] represents the fraction of AH condensed with the PE. Although some drugs upon complexation with EU yield clear aqueous dispersions, it is generally necessary to add an inorganic counterion (i.e. Cl^−^) to increase the aqueous compatibility [1, 2]. Therefore, the composition having fifty mole percent of each counterion (EU(AH)_50_(HCl)_50_) has been used to obtain the results reported in this paper. According to eq. 1, the high degree of counterion condensation between EU and AH produces an increase in the aqueous compatibility of low solubility acid drugs in the range of pH in which solubility is controlled by the undissociated species AH (1). In line with this finding, drug release from such dispersions in bi-compartmental Franz cells has shown that the complexes behave as carriers that slowly release the drug as water is placed in the receptor compartment. In addition, as water was replaced by a NaCl solution to mimic a biological fluid, a rise of release rate was produced. This is a consequence of the diffusion of the salt ions to the sample compartment that generates an ion exchange process with the complex.

Eq. 2. $$[A^{-}{\text{EUH}}^{+}]+\text{NaCl}⇄{\text{EUH}}^{+}+{\text{Cl}}^{-}+A^{-}+{\text{Na}}^{+}$$

This information reveals that (EU(AH)_50_(HCl)_50_) complexes exhibit interesting properties that could be exploited to design drug delivery systems. Hence, the knowledge about the factors that determine the interaction between ionizable acid drugs and the cationic PE is relevant for that purpose.

**Fig. 1.**
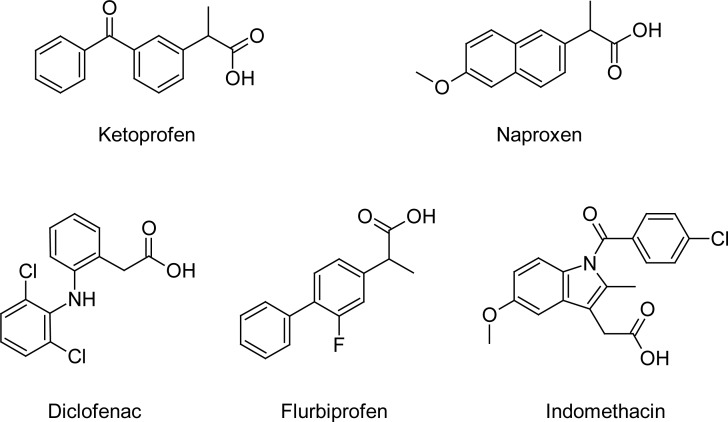
Molecular structure of acidic drugs

On this basis, we report here the description of equilibria and release properties of aqueous systems consisting of a set of five non-steroidal anti-inflammatory drugs (NSAID) complexed with EU. Since the set contains NSAID with and without an alpha methyl group adjacent to their carboxylic moieties (figure 1), the effect of such structural difference on the affinity for the PE is also addressed.

## Results and discussion

Table 1 reports some structural and physico-chemical published data of the NSAID used in the study together with experimentally determined cyclohexane-water (CH/W) partition coefficients.

In line with their structures, the five NSAID exhibit high octanol water (O/W) partition coefficients, low water solubility and acidic groups having the typical strength of aliphatic carboxylic acids. According to equation 1, the stoichiometric drug concentration [AH] in a EU-AH dispersion is given by:

Eq. 3. $$\left[\text{AH}]=(AH)+(A^{-})+({\text{EUH}}^{+}A^{-}]\right)$$

As mentioned earlier, the introduction of *Cl*^−^as a second counterion yields high aqueous compatible systems [1, 2]. In fact, the raise of compatibility of AH, at pHs in which the undissociated species *AH* controls the solubility, results evident. Dispersed complexes exhibit high positive electrokinetic potential (ξ). Table 2 reports these results for the set of dispersions of (EU(AH)_50_(HCl)_50_) at 0.5% of EU complementing those previously reported for EU- Diclofenac [2]. It is clear that EU-NSAID complexes produce clear, stable aqueous dispersions that expand the interval of pH of NSAID compatibility.

To obtain quantitative information about the distribution of species in the aqueous dispersions (EU(AH)_50_(HCl)_50_), the analytical method previously reported in [1] was used. Briefly, the method is based on the selective extraction with cyclohexane of the undissociated *AH* present in the aqueous medium.

In this procedure we calculated the molar proportions of (*AH)*, (*A*^−^*)*, and ([EUH^+^*A*^−^]) at four different concentrations of EU that are reported in table 3 as percentages of each species.

Such results reveal that a remarkably high proportion of AH is condensed with the PE under the form of ion pairs. It is worth noting that the proportion of counterionic condensation in these systems is significantly higher than that exhibited by acid polymetacrylates and model basic drugs [12, 13].

**Fig. 2.**
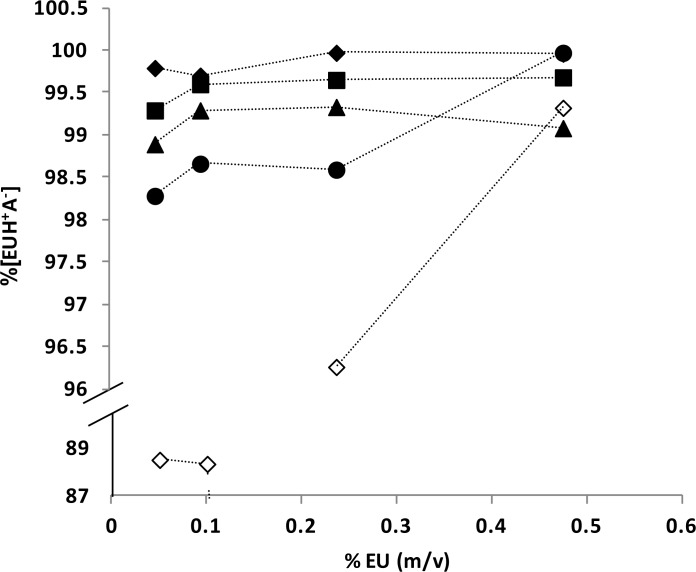
Percentage of [EUH^+^A^−^] in (EU-(AH)_50_(HCl)_50_) complexes at increasing concentrations of EU after partition equilibrium with CH. (▴)F, (▪)I, (♦)D, (•)K, (⋄)N.

On the other hand, Figure 2 shows that the proportion of [EUH^+^*A*^−^] in the complexes of F, N and K was lower than in D and I ones under all conditions assayed except that of K at 0.5% of EU. Since the former group has the methyl group in alpha position, these results suggest that the presence of such moiety affects the counterionic condensation equilibrium.

In this regard, classical description of ion-ion interaction recognizes two relative stable regions, one referred to as a *solvent separated ion pair*, or as a *loose ion pair* and the other referred to as a *contact ion pair,* which is also known as a *tight ion pair* [14].

In the same line, within the framework of the counterion condensation theory of PE, a common point in the theoretical treatments proposed is the recognition of two extreme modes of counterion association with the PE, currently referred to as *loose* and *covalent bonding*. The former is the delocalized confinement of the counterions within a condensation volume in the immediate vicinity of the PE, due only to long range interactions, while the latter is a short range, site-specific interaction [15–17].

Theoretical treatments mainly address the interaction of linear PE with inorganic ions. However, with organic counterions, although the main contribution to the overall interaction arises from the electrostatic attraction, non-electrostatic contributions would also play a role in the association process.

Diffusion of AH from (EU(AH)_50_(HCl)_50_) complexes through a semipermeable membrane was evaluated in bicompartimental Franz cells. To observe the release against a simulated biological fluid, a 0.9% NaCl solution was placed in the receptor compartments of the cells. Therefore, the diffusion of Na^+^ and Cl^−^ to the upper compartment produces an ionic exchange with the complex that yields free *AH* and *ANa* species able to diffuse. Under such conditions the five complexes exhibited a remarkable zero order of release. It is clear that EU-NSAID complexes dispersed in water behave as drug carriers that modulate the drug release in contact with a simulated biological fluid. Therefore, they are potentially useful for designing drug delivery systems of this important drug family. Additionally, in line with the percentages of counterionic condensation observed previously, release rates from F, N and K complexes (figure 3 part a) were clearly higher than those of D and I (figure 3 part b). Therefore, from the present results, it appears that the presence of the alfa methyl group negatively affects the intensity of the interaction between the protonated dimethylamino pendant groups of the PE and the drug carboxylates.

## Conclusions

The determination of the degree of counterionic condensation revealed a remarkable affinity between NSAID and EU. Affinity was higher for members without an alpha methyl group in relation to those bearing such a group.

The complexation with EU expands the interval of pH of NSAID compatibility, which is an interesting finding in the field of drug formulation.

The five complexes behave as drug carriers that modulate the release of the drug in contact with 0.9% NaCl solution, which is a good approach to mimic biological fluids such as tears and wound exudates. Under such conditions they exhibit zero order of release rate and are potentially useful for designing drug delivery systems for topical administration to the eye and wound treatment.

In line with the percentages of counterionic condensation observed, release rates from –(CH_3_)CH-COOH complexes were clearly higher than those of –CH_2_-COOH ones.

Last, the analysis performed on the complexation equilibra among two kinds of NSAID and EU, the resulting uptake of the drugs and the understanding of the release mechanism under conditions of therapeutic interest, provides solid basis to the rational design of delivery systems.

## Materials and methods

### Materials

Poly (butyl methacrylate, 2-(dimethylamino)ethyl methacrylate, methyl metacrilate) 1:2:1 (Eudragit® E100, Pharmaceutical Grade, Rohm, Germany) was a gift from Etilfarma S.A. (Buenos Aires, Argentina), sodium diclofenac (D), indomethacin (I), ketoprofen (K), naproxen (N), flurbiprofen (F), (Pharmaceutical grade, Parafarm, Buenos Aires, Argentina), hydrochloric acid, cyclohexane (CH, Anedra) acetone (PA grade, Cicarelli, Santa Fe, Argentina), sodium chloride (NaCl, PA grade), 1.000 N sodium hydroxide solution (Anedra, San Fernando, Argentina); were also used. Diclofenac was obtained from an aqueous solution of sodium diclofenac acidified with diluted HCl. A white precipitate of acid diclofenac formed was filtered, washed with bidistilled water and dried.

### Preparation of(E U(AH)_50_(HCl)_50_) solid complexes

Before complexation, EU was milled and sieved through 40 and 70 mesh sieves. The equivalents of amino groups per gram of EU (3.10×10^−3^) were assayed by acid base titration. The complexes were prepared by dispersing 1 g of EU in 15 ml of acetone, and the appropriate amount of AH to neutralize 50% of the amino groups of EU. The remaining basic groups of the polymer were neutralized with 1.0N HCl. Then, the solvent was evaporated under vacuum at room temperature.

### Aqueous compatibility

Weighed amounts of (EU(AH)_50_(HCl)_50_) were dispersed in water to obtain clear dispersions having 0.5% (w/v) of EU whose pHs were recorded.

### Electrokinetic potential (ξ)

A Mark II Rank Broders LTD microelectrophoresis apparatus was used with a device of 10 cm between electrodes and 50 ± 0.5V electric field.

### Partition equilibrium with cyclohexane (CH)

Samples of aqueous dispersions of AH complex (EU(AH)_50_(HCl)_50_) at 0.05–0.5% of EU were shake flask partitioned with CH at CH/aqueous dispersions ratio of 2. Concentration of AH in CH was spectrophotometrically assayed at 251, 323, 278, 247 and 273nm for K, I, D, F, and N, respectively, and pH was recorded before extraction and at equilibrium. In the same way the partition equilibrium CH/water of AH was measured to get the true partition coefficient (PCt).

### Drug delivery from(EU(AH)_50_(HCl)_50_)aqueous dispersions at 0.1 % of EU

Experiments were performed in a modified Franz diffusion assembly at 37± 1°C. Semipermeable acetate cellulose membrane (Sigma® 12,000) was placed between the donor and receptor compartments. Samples of 3 ml of each dispersion were placed in the upper compartment. The receptor compartment was filled with 76 ml of 0.9% NaCl solution stirred at 200 rpm with Teflon-coated magnetic stirring bar. At selected times, 4 ml aliquots were withdrawn and replaced by the same volume of receptor medium. Data were corrected for dilution. AH concentration was determined by UV spectroscopy (D=276 nm, I=320 nm, F=248 nm, N=296 nm and K= 260 nm). The assays were done in triplicate.

## Figures and Tables

**Fig. 3a.**
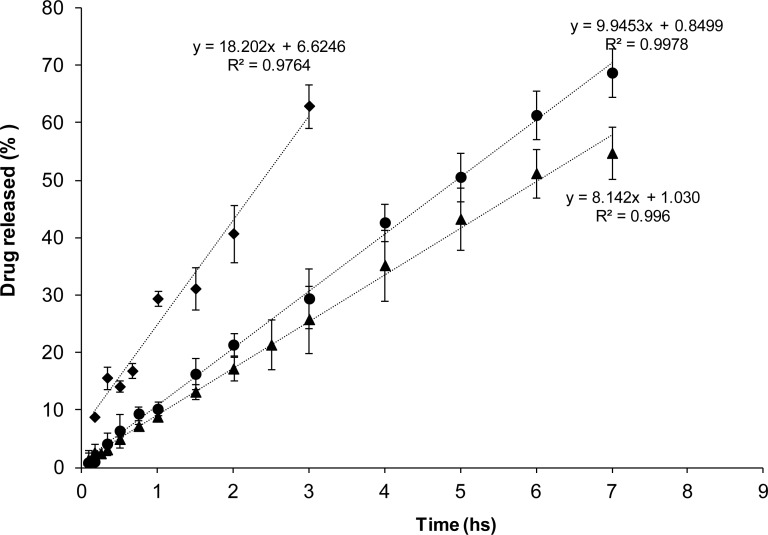
Release profiles of (♦)K, (•)N, (▴)F from complex dispersions (EU(AH)_50_(HCl)_50_) at 0.1% EU, using 0.9% NaCl solution as the receptor medium.

**Fig. 3b.**
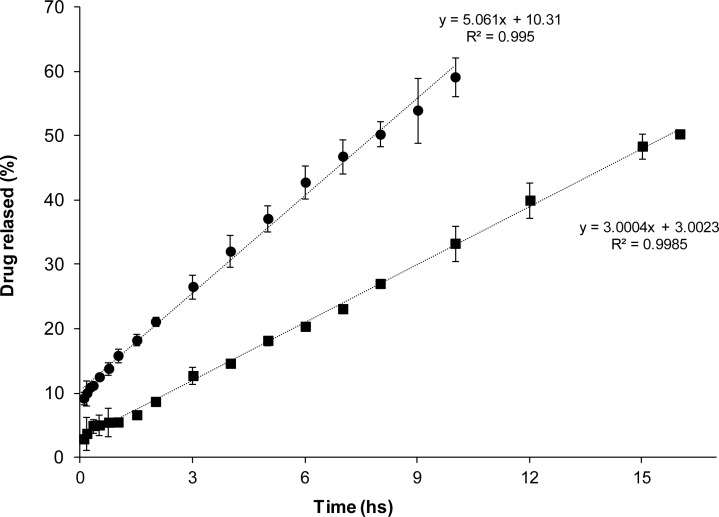
Release profiles of (▪)D and (•)I from complex dispersions (EU(AH)_50_(HCl)_50_) at 0.1% EU, using 0.9% NaCl solution as the receptor medium.

**Tab. 1.** Solubility, pKa, MW, log P and structural change of selected acidic drugs.

**Drugs**	**-CH-COOH X**	**Log P** [11] **O/W**	**Log P CH/W**	**MW**	**pKa** [10]	**Solubility water 25°C (mg/mL)**
Diclofenac (D)	-H	4.40	2.09	296.20	4.5	6.00 10^−3^ [6]
Flurbiprofen (F)	-CH_3_	4.24	1.87	244.26	4.2	2.70 10^−2^ *
Indomethacin (I)	-H	4.27	0.82	357.81	4.5	4.00 10^−3^ [7]
Ketoprofen (K)	-CH_3_	3.12	1.48	254.29	4.8	1.69 10^−1^ [8]
Naproxen (N)	-CH_3_	3.34	0.89	230.26	4.2	3.10 10^−2^ [9]

* The solubility of Flurbiprofen was determined experimentally.

**Tab. 2.** Quotient between the concentration of AH dispersed as (EU(AH)_50_(HCl)_50_) at 0.5% EU and the solubility of AH (mg/ml) at the same pH (Temperature 25°C).

**(EU-(AH)_50_(HCl)_50_) 0.5%EU**	**pH**	**[AH] mg/ml II^a^**	**[AH] mg/ml I^b^**	**Increase of solubility II/I**	**ξ (mV)**
Diclofenac	4.50	1.1 10^−02^	2.31	212	+38.9
Flurbiprofen	3.91	4.2 10^−02^	1.95	47	+44.1
Indomethacin	4.45	7.6 10^−03^	1.73	229	+51.4
Ketoprofen	4.46	2.46 10^−1^	2.16	9	–
Naproxen	4.72	1.16 10^−1^	1.92	17	–

a AH aqueous solubility at the pH of the corresponding complex dispersion;

b Concentration of AH in (EU(AH)_50_(HCl)_50_) at 0.5% EU (w/v).

**Tab. 3.** Species distribution at equilibrium of a set of (EU(AH)_50_(HCl)_50_) (0.5%–0.05%)EU after the partition with CH.

**(EU(AH)_50_(HCl)_50_)**	**0.05% EU**			**0.1% EU**		
	
	**(*AH*) %**	**(*A*^−^) %**	**([EUH^+^*A*^−^]) %**	**(*AH*) %**	**(*A*^−^) %**	**([EUH^+^*A*^−^*]*) %**

Diclofenac	0.06	0.15	99.79	0.08	0.21	99.70
Flurbiprofen	0.17	0.93	98.89	0.09	0.61	99.29
Indomethacin	0.36	0.35	99.29	0.29	0.18	99.60
Ketorpofen	1.50	0.23	98.28	1.19	0.15	98.66
Naproxen	5.29	6.29	88.51	4.92	6.74	88.34

**(EU(AH)_50_(HCl)_50_)**	**0.25% EU**			**0.5% EU**		
	
	**(AH) %**	**(*A*^−^) %**	**([EUH^+^*A*^−^]) %**	**(*AH*) %**	**(*A*^−^) %**	**([EUH^+^*A*^−^]) %**

Diclofenac	0.01	0.02	99.97	0.016	0.07	99.96
Flurbiprofen	0.12	0.55	99.33	0.12	0.79	99.08
Indomethacin	0.19	0.16	99.65	0.14	0.18	99.68
Ketorpofen	0.94	0.43	98.59	1.05	0.48	99.97
Naproxen	0.14	0.23	96.26	0.46	0.50	99.32
